# Joining of Aluminum and CFRP via Laser Powder Bed Fusion: Influence of Experimental Set-Up and Laser Processing on Microstructure and Mechanical Properties

**DOI:** 10.3390/polym15183839

**Published:** 2023-09-21

**Authors:** Sara Nester, Dieter Meinhard, Jochen Schanz, Markus Rettenberger, Iman Taha, Harald Riegel, Volker Knoblauch

**Affiliations:** 1 Materials Research Institute, Aalen University of Applied Sciences, Beethovenstraße 1, D-73430 Aalen, Germany; 2 Laser Application Center, Aalen University of Applied Sciences, Beethovenstraße 1, D-73430 Aalen, Germany; 3 Sustainable Materials in Polymer Engineering, Aalen University of Applied Sciences, Beethovenstraße 1, D-73430 Aalen, Germany

**Keywords:** hybrid joints, fiber-reinforced polymer–metal joining, additive manufacturing, laser powder bed fusion

## Abstract

Additive-manufacturing-based joining methods enable tailored or even functionalized joints and allow for hybridization at small scales. The current study explored an innovative joining method for aluminum cast alloys (AlSi12) with thermoset carbon-fiber-reinforced polymers (CFRPs) via laser powder bed fusion (LPBF). The direct build-up of AlSi12 on a CFRP substrate proved to be challenging due to the dissimilar thermal properties of the considered materials, which led to substrate damage and low joint adhesion. These effects could be overcome by introducing an AlSi12 foil as an interlayer between the two joining partners, acting as a thermal barrier and further improving the AlSi12 melt wettability of the substrate. Within LPBF, the energy input in the form of volumetric laser energy density influenced both the porosity of the fused layers and the formation of thermally induced stresses due to the high cooling rates and different thermal expansion properties of the materials. While the AlSi12 volume density increased with a higher laser energy input, simultaneously increasing thermal stresses caused the debonding and deformation of the AlSi12 foil. However, within a narrow processing window of laser parameters, the samples achieved remarkably high shear strengths of τ > 20 MPa, comparable to those of conventional joining methods.

## 1. Introduction

Hybrid joints for lightweight applications, e.g., aviation and automotive components, enable the local modification and adaption of component properties through the combination of metals and polymer composites [1,2]. However, manufacturing high-performance joints of metals and fiber-reinforced polymers is challenging due to their physico-chemical incompatibility and low mutual solubility [3,4]. Conventional and reliable joining methods, e.g., adhesive bonding or mechanical fastening, suffer limited design flexibility and geometrical freedom [1,5]. Additive manufacturing, as a sequential layer-by-layer process for material build-up, enables the production of three-dimensional, near-net-shape components based on a digital model [6]. Therefore, research is conducted on various additive-manufacturing-based joining methods, which increase the freedom of shape, enable a tailored or even functionalized joining area and allow for targeted hybridization across all scales [7,8,9].

Several studies within this field have focused on the modification and progression of fused deposition modeling (FDM) for polymers on metal substrates. In 2016, Amancio-Filho and Falck developed the “AddJoining” method for the manufacturing of layered metal–polymer hybrid structures via FDM (German patent DE 10 2016 121 267 A1). Since then, the technology was used for various material combinations, e.g., single-lap joints from acrylonitrile butadiene styrene on Al 2024 substrates with a shear strength of 5.3 ± 0.3 MPa [1,10], as well as for alternating layers of unreinforced and carbon-fiber-reinforced polyamide 6 on Al 2024 substrates, resulting in 21.9 ± 1.1 MPa shear strength [11].

Other research groups combined additive manufacturing process strategies for the build-up and simultaneous joining of polymers with metals. Matsuzaki et al. formed resin molds via FDM and filled them with copper via electroforming (electrolytic copper plating). Based on component design, the FDM-printed parts functioned as structural units within the hybrid [12]. Furthermore, Oliveira et al. [13] produced hybrids from polycarbonate and AlSi10Mg completely via additive manufacturing. They prepared AlSi10Mg parts with submillimeter-sized surface structures using laser powder bed fusion (LPBF), and these served as substrates for the subsequent FDM printing of polycarbonate, resulting in a joint strength of 10.8 ± 0.6 MPa.

As LPBF involves the application and bonding of the initial powder layer on build plates, joining via direct deposition on conventionally manufactured substrates has already been studied for dissimilar metal [14,15] and metal–ceramic [16,17] composites. In the literature, the following challenges were identified for multimaterial LPBF: different thermal properties of the used materials, the formation of brittle intermetallic phases at the interface and the wettability of the melt on the substrate surface [16,18]. These limitations could be overcome by the introduction of specific interlayer materials for a variety of material combinations [14,15,16,17]. Following this approach, Azizi et al. introduced a Sn3Ag4Ti interlayer at the surface of pyrolytic graphite, which was used as a build plate in LPBF with stainless steel (316L) to enable the rapid single-step production of heat sinks or exchangers [19,20].

Regarding the multimaterial LPBF of metal–polymer composites, Chueh et al. [7] studied the side-by-side laser processing of Cu10Sn and polyamide powders in a single powder bed. However, the fusion of the Cu10Sn powder required a high laser energy input, which led to the overheating and decomposition of polyamide, causing the resulting carbon deposits to interrupt the melting of subsequent layers. Therefore, the dissimilar powders were laser-scanned with a specific gap to overcome undesirable material interactions.

Due to their elevated melting and processing temperatures, additive manufacturing processes for metals on thermally sensitive polymer substrates are challenging and thus hardly investigated for hybridization. However, reinforcement materials for polymer composites are usually more resistant to elevated temperatures, e.g., carbon fibers with a decomposition temperature of 3650 °C in the absence of oxygen [21]. Therefore, Gibson et al. used Ni-coated carbon fiber woven fabric, on which they built Ti6Al4V layers via LPBF. A cross-section analysis revealed that the Ti6Al4V melt penetrated about half of the fabric thickness, thereby forming a metal–matrix composite [22]. In the following step, the non-filled fabric side was infiltrated with polymer resin to obtain a hybrid metal/CFRP composite with a functionally graded interface [9].

The current study deals with the build-up of metal layers on carbon-fiber-reinforced polymer (CFRP) substrates through LPBF as a method for additive manufacturing. The aim was to develop an innovative joining method for flexible and functional hybrid composites, including form-fit joints, direct connections or edge reinforcement with complex geometry and additional internal features such as cable or cooling channels. Fundamental investigations on the feasibility of the proposed method were examined using AlSi12 powder deposited on thermoset CFRP substrates. Prior to LPBF, the thermally unstable polymer matrix was removed via laser ablation in order to apply and melt the AlSi12 powder directly on the exposed carbon fibers. Within the following experiments, an AlSi12 foil was introduced into the substrate set-up to overcome the thermal differences in the used material combination. In order to detect the processing window for LPBF, the microstructure of the resulting hybrids was examined via light microscopy in top-view as well as cross-section images. In addition, thermogravimetric and thermo-mechanical analyses of the individual materials were carried out to allow for a deeper understanding of their behavior during LPBF. Moreover, a method for quasi-static mechanical tensile testing was developed to evaluate the shear strength of the CFRP/AlSi12 joint. In order to introduce the forces in this specific interface, a counterbody for the AlSi12 volume was used according to the “mortise-and-tenon” geometry by Silva et al. [23].

## 2. Materials and Methods

### 2.1. Materials

**Figure 1:**
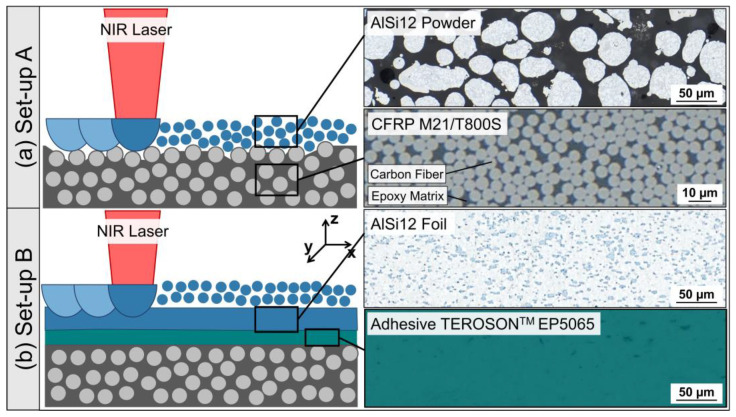
Substrate set-ups for LPBF: (**a**) set-up A: direct build-up of AlSi12 powder on laser-pre-treated CFRP M21/T800S, (**b**) set-up B: AlSi12 powder on AlSi12 foil/CRFP substrate, adhesively bonded with TEROSON^TM^ EP5065; light microscope (LM), bright field (BF).

The thermoset-based CFRP M21/T800S (Hexcel Corporation) is a multiaxial, quasi-isotropic laminate from eight unidirectional prepreg layers in a [0/45/90/-45]_s_ stacking sequence. TORAYCA^®^ T800S fibers of 5 µm diameter served as reinforcement for the epoxy-based matrix system Hexcel HexPly^®^ M21; see Figure 1a. Laminates of 2 mm thickness were hot-pressed and cured at 180 °C and 7 bar. Gas-atomized AlSi12 powder (ECKA Granules Germany GmbH, Velden, Germany) with the following particle size distribution was used for the LPBF process: d_10_ = 24.1 µm, d_50_ = 38.7 μm and d_90_ = 59.7 μm. The pre-alloyed spherical Al powder with 11.9 wt% Si showed a dendritic cast microstructure; see Figure 1a. Moreover, an AlSi12 foil with a 0.2 mm thickness and a melting range of 575–585 °C was used (voestalpine Böhler Welding Group GmbH, Düsseldorf, Germany). The microstructure of the foil presented Si precipitations in an Al matrix; see Figure 1b. The CFRP substrate and AlSi12 foil were bonded with the two-componential epoxy and amine-based adhesive TEROSON^TM^ EP5065 (Henkel AG & Co. KGaA, Düsseldorf, Germany); see Figure 1b. Prior to joining, the adherents were cleaned with acetone. The adhesive thickness of 0.2 mm was controlled by mixing 1 vol% micro-glass beads (Sigmund Lindner GmbH, Warmensteinach, Germany) with a particle size of 180–212 µm into the adhesive.

### 2.2. Substrate Set-Up and Preparation

LPBF is based on the layer-by-layer fusion of powder on a substrate with the help of laser energy. First, LPBF experiments were carried out by layering and laser melting AlSi12 powder directly on the CFRP substrate (set-up A); see Figure 1a. In advance, near-infrared (NIR) laser pre-treatment was carried out to ablate the surface matrix layer from the CFRP substrate, herewith exposing carbon fibers targeting an improved bonding with the AlSi12 melt. The CFRP pre-treatment was conducted according to previous studies, addressing the influence of laser pulse parameters on the matrix and carbon fiber ablation behavior [24] and correlating them with the bonding strength of hybrid CFRP/Al joints [25]. Detailed descriptions of the applied laser system and laser parameter with an areal energy density of 0.37 J/mm^2^ for the matrix ablation are presented in [26]. After laser ablation, the CFRP substrate was adhesively bonded to a steel build plate with an instant adhesive (Pattex Ultra Gel).

**Figure 2:**
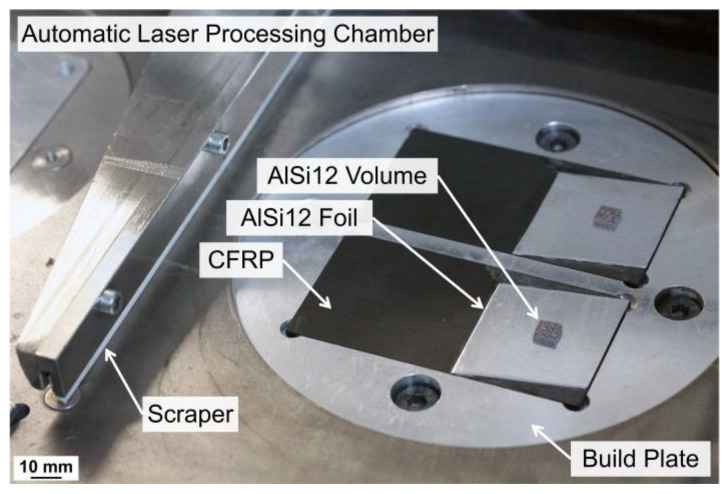
Automatic laser processing chamber used to manufacture test specimens for mechanical tensile strength analysis.

As a result of the findings from set-up A, the substrate structure was modified for further LPBF experiments according to set-up B; see Figure 1b. An AlSi12 foil was laminated on the CFRP as an interlayer between the CFRP and the additively printed AlSi12 powder. In this case, the CFRP and AlSi12 foil were cleaned with acetone and adhesively joined, but the foil should be integrated during CFRP manufacturing for improved process efficiency. Prior to LPBF, the substrates were inserted in steel build plates with an instant adhesive; see Figure 2.

### 2.3. Laser Powder Bed Fusion

LPBF was conducted with a TruFiber 1000 single-mode fiber laser (TRUMPF Laser- und Systemtechnik GmbH, Ditzingen, Germany) working in an NIR wavelength of λ = 1075 nm with a maximum laser power of P_max_ = 1000 W and a focal diameter of d_f_ = 46 µm; see Table 1. For laser scanning, the 2D SCANLAB intelli*SCAN* 30 optical galvanometer scan system (SCANLAB GmbH, Puchheim, Germany) was used.

LPBF of the AlSi12 powder was conducted in the absence of oxygen in a laser processing chamber, which was flooded and operated with argon. The experiments for substrate set-up A were executed with a flexible, manual laser processing chamber for a small sample size with a low powder usage below 13 cm^3^. Within this set-up, the Ø 23 mm build plate and the powder coating were conducted manually. For substrate set-up B, the experiments were scaled up to an automatic laser processing chamber providing motorized build plate control and powder application; see Figure 2. The build plates had a Ø 120 mm diameter and a building area of 76 × 76 mm^2^. With a maximum build height of 25 mm and a powder supply of 280 cm^3^, this chamber enables the production of larger samples. Both processing chambers were designed and manufactured at Aalen University specifically for research in material science.

Regardless of the substrate set-up, the constant LPBF parameters involved the laser beam diameter, layer thickness and hatch distance; see Table 2. The laser beam was operated in continuous-wave mode and defocused from the focal diameter d_f_ = 46 µm to d_f5 mm_ = 200 µm to enlarge the laser spot area while reducing its intensity. The hatch distance was adjusted from H_s_ = 46 µm to H_s_ = 200 µm between set-ups A and B to reduce the hatch overlap from 77% to 0%. The scan velocity was constant for substrate set-up A and varied between V_s_ = 400 and 700 mm/s for substrate set-up B. The adjustment of the laser power for each set-up resulted in higher volumetric energy densities (VEDs) for substrate set-up B, calculated using the equation given by Kumar et al. [27]. The scan fields for substrate set-up A with an area of 7 × 7 mm^2^ were laser-scanned in a forward–backward pattern, which was divided into a chessboard pattern with four scan fields of 2.75 × 2.75 mm^2^ and a 0.25 mm overlap for substrate set-up B.

### 2.4. Thermal Analysis

A thermogravimetric analysis (TGA) was conducted using the thermobalance type TGA/DSC 3^+^ (Mettler-Toledo GmbH, Gießen, Germany) with an accuracy of 1 µg to analyze the degradation behavior of the adhesive and the CFRP. Prior to TGA, specimens were cut with a diamond band saw 300 CL (EXAKT Advanced Technologies GmbH, Norderstedt, Germany) and placed in a 70 µL alumina crucible. TGA measurements were performed over a temperature range from 30 to 850 °C, with a purge gas flow of 50 mL/min and a constant heating rate of 20 K/min. Nitrogen (N_2_) was used as the purge gas up to 650 °C, above which the purging gas was switched to oxygen (O_2_).

A thermo-mechanical analysis (TMA) of the adhesive was performed with the analytical system TMA/SDTA 2+ (Mettler-Toledo GmbH, Gießen, Germany), with an accuracy of 0.5 nm. For sample preparation, the adhesive was molded at a thickness of 2.3 mm and cut to an area of 5 × 5 mm^2^. Thermal expansion in the thickness direction of the adhesive was measured under a 20 mN static compressive force with a standard stamp. The measurement was conducted at a 2 K/min constant heating rate over a temperature range of 30 to 300 °C in an inert N_2_ atmosphere. The TMA of the AlSi12 foil was conducted using a TMA 402 F1/F3 Hyperion dilatometer (Netzsch-Gerätebau GmbH, Selb, Germany) with a vertical clamping holder to apply a 20 mN static tensile force. Prior to TMA, foil samples with 6 × 30 mm^2^ were cut and clamped over a 14 to 15 mm distance. A constant heating rate of 2 K/min was applied over a temperature range of 30 to 500 °C in an inert N_2_ atmosphere.

### 2.5. Mechanical Characterization and Fractographic Investigations

**Figure 3:**
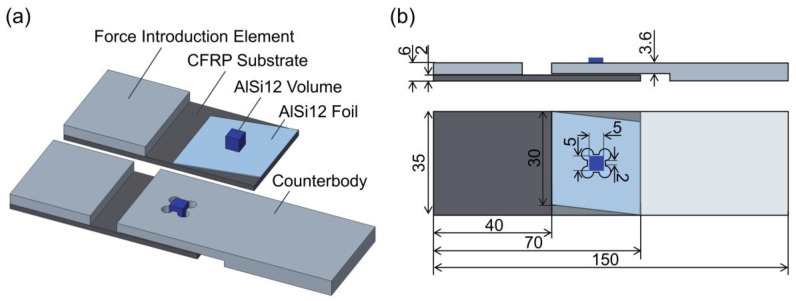
Concept of mechanical strength analysis: (**a**) specimen set-up for force introduction at the AlSi12 volume/AlSi12 foil interface, (**b**) specimen geometry and dimensions.

Substrate set-up B enabled the scale up of the process and the manufacture of specimens for a shear strength analysis in reference to DIN EN 1465:2009 [28]. However, the sample configuration was significantly modified to apply shear forces at the interface between the AlSi12 volume and AlSi12 foil. The “mortise-and-tenon” joints introduced by Silva et al. [23] are based on the interlocking of additively manufactured tenons with corresponding cut-outs in a joining partner. This principle was applied by using a steel counterbody with a fitting mortise; see Figure 3a. The CFRP, adhesive and steel joining partner geometries were significantly oversized compared to the AlSi12 volume with dimensions of 5 × 5 × 5 mm^3^ to avoid their failure in shear strength testing; see Figure 3b. To ensure a smooth powder coating over the AlSi12 foil during LPBF, the foil edge was angled by 10° to the coating edge of the powder scraper; see Figure 3b. To prevent the deflection and bending of the “mortise-and-tenon” system during mechanical testing, a frame similar to DIN 65148:1986 [29] was manually bolted on the specimens. To compensate for the asymmetry of the single-lap joints and ensure authentic shear failure, cap strips were bonded to the CFRP substrate and integrated in the steel counterbody. Mechanical testing was executed using the Schenck RSA100 universal testing machine under normal temperature and pressure with a 100 kN load cell at a traverse speed of 1.5 mm/min and a pre-load of 100 N. The test protocol was terminated when the maximum shear strength decreased by 90%. The shear strength values of five samples per laser parameter were averaged. A fracture analysis was conducted by means of top-view images with an Axio Zoom.V16 light microscope (Carl Zeiss AG, Oberkochen, Germany).

### 2.6. Macro- and Micro-Structural Characterization

Documentation of the specimens’ macroscopic structure after LPBF was carried out via top-view bright-field light microscopy with Axio Zoom.V16 (Carl Zeiss AG, Oberkochen, Germany). A microstructural analysis was performed on materialographic cross-sections. Hence, the specimens were embedded in an epoxy-based mounting system (Struers GmbH, Willich, Germany) to preserve their condition. After curing, the samples were cut perpendicular to the LPBF scanning direction and re-mounted for mechanical grinding and polishing with the automatic preparation system Tegramin 30 (Struers GmbH, Willich, Germany). Large-scale light microscope images of the entire specimen cross-section were generated with Axio Imager Z.2 Vario (Carl Zeiss AG, Oberkochen, Germany). For improved contrast between the adhesive and the CFRP matrix, the cross-section images were post-processed to display the adhesive in green color.

## 3. Results

### 3.1. Substrate Set-Up A

**Figure 4:**
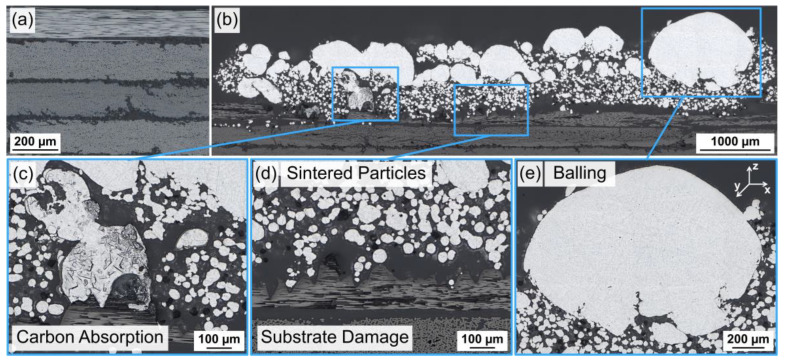
Substrate set-up A: (**a**) cross-section [0/45/90/-45]_s_ layers of CFRP M21/T800S (as-received), (**b**) cross-section overview for VED = 14.4 J/mm^3^ (V_s_ = 400 mm/s and P = 65 W), (**c**) carbon absorption in re-solidified AlSi12, (**d**) surface-near CFRP substrate damage and substrate-near sintered AlSi12 particles, (**e**) re-solidified AlSi12 spheres; LM, BF.

The variation in the different process parameters, such as the laser power, did not render reproducible adhesion and liable bonding between the AlSi12 powder and CFRP substrate. Hence, the results are summarized using the laser parameter VED = 14.4 J/mm^3^ (V_s_ = 400 mm/s and P = 65 W) as an example. The cross-section in Figure 4b–e was cut transverse to the laser scanning direction, corresponding to the Y-axis in Figure 4e. LPBF of ten powder layers, each with a 100 µm thickness, caused critical changes within the CFRP compared to its initial state in Figure 4a. Matrix decomposition and fiber fracture led to a partial removal of the first laminate layer to a depth of 250 µm. Close to the substrate, AlSi12 particles were mainly in a loose or partially sintered state up to a 300–400 µm build height, which corresponds to 3–4 powder layers in LPBF; see Figure 4d. In contrast, the area in Figure 4c indicated melted AlSi12 powder with absorbed carbon particles/fibers at the carbon fiber interface. Upper AlSi12 areas showed re-solidified melt spheres (balling) with a diameter of up to 1 mm and a fine dendritic cast microstructure.

### 3.2. Optimized Substrate Set-Up B

**Figure 5:**
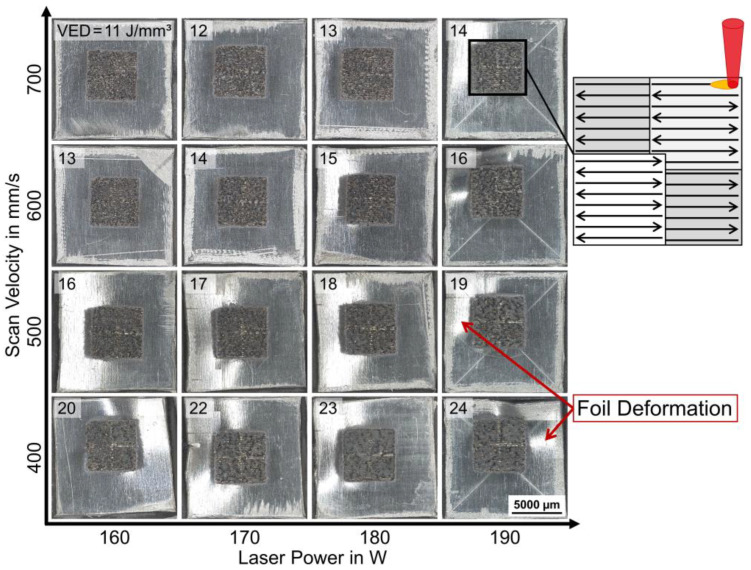
Substrate set-up B: top-view images of AlSi12 volume on AlSi12 foil sections, parameter study with P = 160–190 W and V_s_ = 400–700 mm/s, resulting VED = 11–24 J/mm^3^ (upper left corner in each tile image) with chessboard scanning strategy, LM, BF.

Substrate set-up B used a laminated AlSi12 foil on the CFRP surface as a substrate interlayer for LPBF. A parameter study with VED = 11–24 J/mm^3^ was conducted with variations in laser power and scan velocity; see Figure 5. The top-view images in Figure 5 show that the integration of the AlSi12 foil allowed for the build-up of AlSi12 layers in LPBF for all studied values of VED = 11–24 J/mm^3^. The solidified AlSi12 volumes on top of the AlSi12 foil had a porous surface structure, where the chessboard scanning pattern was visible. With increasing VED > 14 J/mm^3^, the AlSi12 foils exhibited deformation, which was detectable by means of light reflections in the periphery foil areas; see Figure 5.

**Figure 6:**
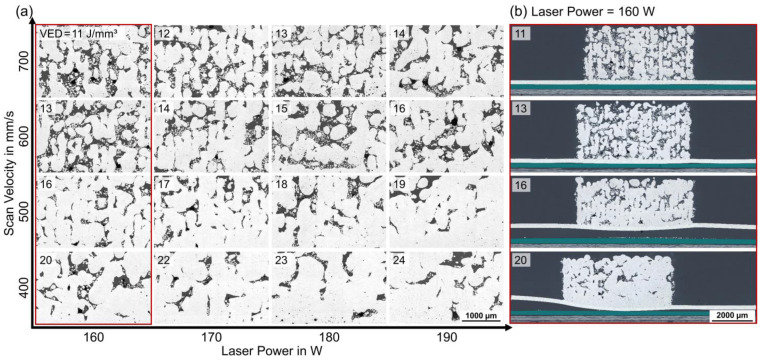
Substrate set-up B, cross-section images, parameter study (P = 160–190 W, V_s_ = 400–700 mm/s) with chessboard scanning strategy (**a**) AlSi12 volume, (**b**) Full-area images of red framed specimens (P = 160 W and V_s_ = 400–700 mm/s): AlS12 volume, AlSi12 foil, adhesive TEROSON^TM^ EP5065 (green) and CFRP M21/T800S, LM, BF.

Cross-sections of the specimens were cut and mounted perpendicular to the laser scanning direction of the chessboard scan strategy in LPBF (Figure 5). The results in Figure 6 enabled in-depth microstructural analyses of the density and pore formation in the AlSi12 volume, as well as of the debonding and deformation of the AlSi12 foil. Segmentation and quantification of the cross-section images regarding solid areas and pores revealed density values of ρ_%_ = 67–91% for the AlSi12 volume. The samples showed an increasing density and a reduced lack of fusion porosity at elevated VED, due to an increasing laser power or a decreasing scan velocity; see Figure 6a. Samples with a higher porosity revealed solid structures as narrow lines at a width of about 200 µm, corresponding to the hatch spacing of the laser beam. Specimens with higher VED > 18 J/mm^3^ fused in the horizontal direction and overcame the linear AlSi12 structures, while the proportion of small round pores increased simultaneously. Samples with low AlSi12 foil deformation with P = 160 W and V_s_ = 400–700 mm/s were selected for full-area cross-section images in Figure 6b. At higher scan speeds, the bonding between the AlSi12 foil and adhesive layer was intact, while a reduction in the scan velocity caused partial to full debonding as well as significant deformation of the AlSi12 foil.

### 3.3. Thermal Analysis

**Figure 7:**
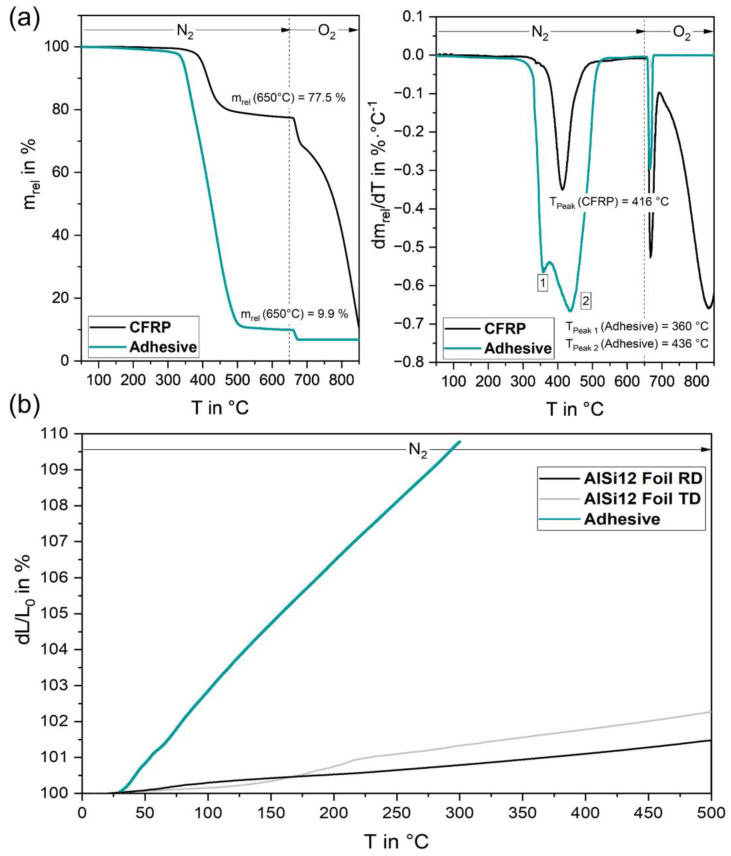
Thermal analyses: (**a**) TGA for CFRP M21/T800S and adhesive TEROSON^TM^ EP5065, (**b**) TMA for AlSi12 foil in rolling direction (RD) and transverse direction (TD) as well as adhesive TEROSON^TM^ EP5065.

TGA of the used CFRP showed that matrix degradation started at about 325 °C under an inert N_2_ atmosphere, reaching a plateau at 650 °C after 22.5% mass loss of the sample; see Figure 7a. The maximum degradation rate was observed at T_Peak_ = 416 °C according to the onset point method. At a subsequent measurement in an oxygen-flooded environment in the range of 650–850 °C, the remaining components were oxidized and converted into H_2_O and CO_2_, which resulted in further mass loss leaving back char and other carbon fiber residues of around 15% of the total mass. TGA of the adhesive indicated significant mass loss starting at 300 °C with maximum degradation rates at T_Peak1_ = 360 °C and T_Peak 2_ = 436 °C reaching a plateau at 500 °C after 90% mass loss; see Figure 7a.

TMA of the epoxy adhesive was conducted from 30 to 300 °C. The results in Figure 7b show an average coefficient of thermal expansion (CTE) of about 370 × 10^−6^/K resulting in about 10% thermal expansion when heated to 300 °C. Thermal expansion of the AlSi12 foil was measured both in the rolling direction (RD) from the foil manufacturing process and in the transverse direction (TD). In the rolling direction, an average CTE of 30 × 10^−6^/K was observed, whereas the foil had a higher average value of 48 × 10^−6^/K in the transverse direction. In general, the foil exhibited significantly lower thermal expansion than the adhesive, reaching a linear elongation of <1.5% at 300 °C and <2.5% when further heated up to 500 °C.

### 3.4. Shear Strength of CFRP/Al Joints and Fracture Patterns

**Figure 8:**
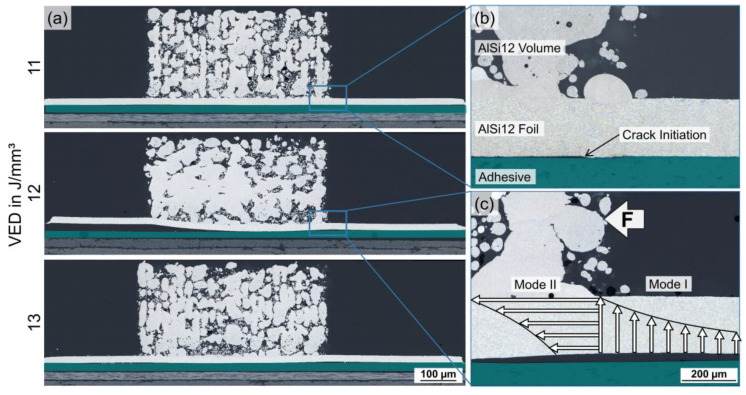
Microstructural analyses of laser parameters for mechanical characterization: (**a**) cross-sections of VED = 11–13 J/mm^3^, V_s_ = 700 mm/s and P = 160–180 W; (**b**) crack initiation at the AlSi12 foil/adhesive interface; (**c**) failure mechanism in mechanical strength analysis; LM, BF.

Based on the processing window determined within the parameter study for substrate set-up B, mechanical characterization was carried for samples with the following laser parameter settings: VED = 11–13 J/mm^3^ (V_s_ = 700 mm/s and P = 160–180 W). The microstructural analysis of these parameters in Figure 8a revealed debonding of the AlSi12 foil for VED = 12 J/mm^3^. This was related to the decentral positioning of scan fields on the AlSi12 foil sections (Figure 5) due to the insufficient measuring precision of the scanner head position prior to LPBF, which resulted in a reduction in the underlying adhesive bond area.

**Figure 9:**
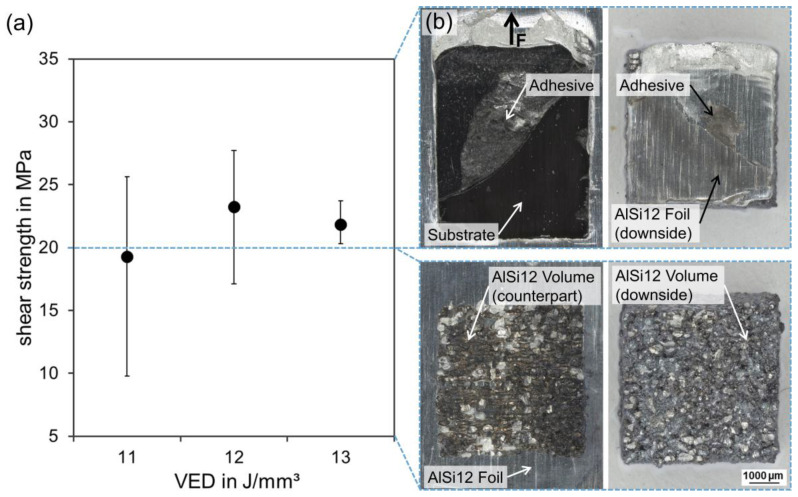
Mechanical characterization: (**a**) shear strength average for VED = 11–13 J/mm^3^ with minimum and maximum values (error bars), (**b**) exemplary fracture patterns for different shear strength regimes; LM, BF.

During mechanical testing, force was applied transverse to the laser scanning direction of the AlSi12 volume. In order to determine the shear strength of the hybrid joints, the maximum force values were related to the bonding area between the AlSi12 volume and foil (5 × 5 mm^2^). Figure 9a shows the averaged shear strength values of five individual measurements for each laser parameter, as well as the minimum and maximum values from the test series (error bars). Samples prepared with VED = 11 J/mm^3^ had an average shear strength of τ = 19.3 MPa, with a minimum of 9.8 MPa and a maximum of 26.0 MPa. For VED = 12 J/mm^3^, the shear strength increased up to τ = 23.3 MPa, with a value range of 17.1–27.7 MPa. The shear strength level remained constant for VED= 13 J/mm^3^ with τ = 21.8 MPa, while the value range decreased significantly to 20.3–23.7 MPa. A post-mortem analysis of the broken composite samples demonstrated two significantly different fracture mechanisms according to the achieved shear strength values. Specimens with shear strength values τ < 20 MPa mainly exhibited adhesive failure at the AlSi12 volume/foil interface along the applied shear plane, while melting lenses as counterparts of the AlSi12 volume were visible on the AlSi12 foil; see Figure 9b. Specimens with τ > 20 MPa showed cohesive failure within the AlSi12 foil, whereas fracture occurred along the edge of the AlSi12 volume facing away from the tensile direction. Since the counter stresses were the highest in the AlSi12 volume, cracks propagated along its edges. The resulting displacement of the AlSi12 volume along the force direction caused adhesive fracture at the AlSi12 foil/adhesive interface. VED = 13 J/mm^3^ achieved exclusive failure through the thickness of the AlSi12 foil at τ > 20 MPa, marking the threshold for reproducible and liable welding at the AlSi12 volume/foil interface.

## 4. Discussion

### 4.1. Substrate Set-Up A: Challenges and Optimization

As the results for substrate set-up A showed, different thermal properties and laser absorption coefficients of the used materials had a critical influence on the CFRP substrate integrity and the melting behavior of the AlSi12 powder and, therefore, on their bonding. The TGA findings related to the investigated CFRP are in accordance with the observations reported by Tranchard et al. for a similar CFRP [30]. Hence, the decomposition temperature (T_Peak_ = 416 °C) of the thermally sensitive polymer matrix was significantly lower than the melting point of the AlSi12 powder (T_m_ = 577 ± 1 °C [31]). In addition, the aluminum required a high laser energy density in LPBF due to its low absorption for NIR and its high thermal conductivity [32]. The used laser parameter (VED = 14.4 J/mm^3^) corresponded to an areal energy density of 1.44 J/mm^2^, which exceeded the threshold for matrix ablation (0.37 J/mm^2^) for the used CFRP as determined by Schanz et al. [24]. The resulting matrix decomposition products generated a gas recoil pressure, which removed the applied AlSi12 powder layer from the CFRP surface. The underlying carbon fibers with a high NIR absorptivity [33] fractured when heated up due to their contraction by negative thermal expansion along the fiber direction (between −0.1 and −0.7 × 10^−6^/K [21]).

As the AlSi12 powder was repeatedly removed from the CFRP surface, the powder layer thickness gradually increased after each scan. The increasing heat capacity of the thicker powder layer reduced the substrate near temperatures to a level where the decomposition of the matrix material, as the most thermally sensitive component, was prevented. Chueh et al. used the same effect in side-by-side LPBF of metal–polymer hybrids to overcome mutual material degradation by processing the powders with a specific gap as a thermal barrier [7]. Consequently, the AlSi12 powder remained on the CFRP surface and fused under the formation of balling, which Zhang et al. attributed to the high surface tension of the aluminum melt, attaining the energetically most favorable state of a sphere [34]. Moreover, Eustathopoulos et al. showed that the passivating oxide layer on aluminum particles prevented liquid-phase equilibrium and reduced melt fluidity when T < 860 °C [35]. Therefore, according to the literature, aluminum melt exhibits contact angles > 90° on graphite substrates at short contact times and moderate temperatures [35,36,37]. As a result, no reproducible wetting and bonding of the AlSi12 melt on the carbon fibers could be achieved in LPBF within substrate set-up A.

The described challenges of a direct processing strategy (substrate set-up A) required a thermal barrier between the high temperatures in LPBF and the thermally degradable polymer matrix of the CFRP. As the current study focused on the vertical build-up and simultaneous bonding of AlSi12 on CFRP, a solid interlayer was integrated at the substrate surface. In addition, depending on the material of the interlayer, the wettability and adhesion of the hybrid joint could be significantly improved. This interlayer approach was already successfully implemented for other material combinations in multimaterial LPBF, such as dissimilar metals [14], metal–ceramic [16] or metal–graphite composites [20]. With regard to metal–polymer composites, this was confirmed through the findings of Rezzoug et al., who modified CFRP with a superficial aluminum mesh layer to increase the adhesion strength of metal coatings in thermal spraying [38]. Accordingly, substrate set-up B was developed, which involved the lamination of an AlSi12 foil to the CFRP prior to LPBF.

### 4.2. Substrate Set-Up B: Microstructure, Thermal and Mechanical Properties

The laser parameter study with substrate set-up B revealed the potential of LPBF as a joining method for hybrid composites through the direct build-up of AlSi12 on top of a modified AlSi12 foil/CFRP substrate. The density and pore formation of the built AlSi12 volume correlated with the applied VED with variations in laser power and scan velocity. This corresponded to Louvis et al. stating that those two parameters mainly determine melt pool properties through laser intensity and exposure time per increment [39]. The lack of fusion porosity, binding defects and balling effects between single layers and lines were therefore reduced with increasing VED, which is attributed to increased melt pool dimensions and reduced melt viscosity in the literature [40,41]. These interrelationships became obvious when linear AlSi12 structures fused, as the theoretical hatch overlap of 0% was exceeded with higher melt pool dimensions. However, the literature shows a positive influence of a low beam overlap due to the reflective behavior of the solid compared to the powder bed [39]. Visible hydrogen porosity in the form of small round pores, which increased with increasing VED, resulted from the hygroscopic properties of aluminum. According to Weingarten et al., this is caused by higher cooling rates promoting the incorporation of vaporized hydrogen in the melt [42].

Since density increased in line with increasing VED, the proportion and quality of the weld bonds at the AlSi12 volume/foil interface also improved. However, the deformation and partial debonding of the AlSi12 foil from the substrate started at elevated VED, leading to an opposing trend when correlating VED with the remaining bond area between the AlSi12 foil and adhesive. This was due to the increasing heating and cooling rates in LPBF, leading to inhomogeneous temperature profiles in the powder bed and the formation of residual stresses during re-solidification as described by Jiang et al. [43]. Since the residual stresses in the AlSi12 volume and foil exerted shear forces at the adhesive interface, small cracks formed at the scan field edges, which propagated in- and outwards with increasing VED; see Figure 8b. According to Jiang et al., thermal stresses accumulate at the scan field edges in LPBF, where the cooling rates are the highest [43]. The deformation of the already debonded AlSi12 foil is further amplified by the mechanical impact of the scraper movement during subsequent powder re-coating processes.

The high thermal conductivity of the AlSi12 powder and foil led to increasing temperatures and thus to the thermal expansion of the underlying substrate layers. Since the adhesive and CFRP polymers were both epoxy-based, they exhibited similar thermal expansion [44]. Accordingly, their interfacial bonding remained intact. However, the TMA results of the AlSi12 foil and the adhesive demonstrated significant differences in their thermal expansion behavior. Pramanik et al. noted that mismatched thermal expansion properties in dissimilar material bonds generate interfacial shear stresses that could potentially lead to joint failure [2]. Additionally, the thermal degradation of the adhesive above 325 °C supported the debonding of the AlSi12 foil due to material loss.

Thermally induced stresses and decomposition became more pronounced at higher VED, leading to significant debonding and deformation of the AlSi12 foil above a threshold value of VED > 14 J/mm^3^. However, parameter combinations with VED ≤ 13 J/mm^3^ had a negligible degree of debonding, which allowed for the quantification of their adhesion strength via mechanical testing. The AlSi12/CFRP joints within the process window achieved shear strength values comparable to those of other additive-manufacturing-based joining methods for metal–polymer hybrids found in the literature [11,13,20]. Moreover, the shear strength even competed with that of conventional joining methods, e.g., Al/CFRP bonds with the same epoxy-based adhesive studied by Schanz et al. [25].

In accordance with the microstructure, the mechanical strength correlated with the laser parameters, reaching superior adhesion at the AlSi12 volume/foil interface with higher VED. When the structural integrity of the AlSi12 volume/foil weld bonds reached τ > 20 MPa, cohesive failure of the AlSi12 foil occurred due to the force introduction into the volume body and resulting stress concentrations at its edge; see Figure 8c. The debonding of the AlSi12 foil at the scan field edges created a multi-axial stress state in this region involving normal and shear stresses. The combination of stress modes I and II exceeded the tensile strength of τ = 185 ± 2 MPa of the AlSi12 foil, which was why failure occurred through the cross-section of the foil.

In 2020, Laursen et al. found that density levels < 95% were critical for the mechanical properties of LPBF volumes from AlSi10Mg [45], as porosity-induced stress concentrations cause crack formation resulting in unexpected fracture during application [46]. However, post-treatment strategies for pore reduction in LPBF volumes, such as hot isostatic pressing, require high temperatures of 350–500 °C [47], which would lead to the decomposition of the thermally sensitive polymer matrix in the hybrid composite. An increase in the bulk density of AlSi12 volumes could be enabled by an increase in VED at a certain distance from the substrate, which was demonstrated by Gibson et al. for LPBF of Ti6Al4V on carbon fiber woven fabrics [22]. Based on the current fracture mechanism, decreased densities < 80% have no critical impact on the bond strength of the hybrid joint.

## 5. Conclusions

The aim of this study was to develop an innovative joining method for flexible and functional hybrid metal–polymer composites through additive manufacturing via LPBF, focusing on the fusion and simultaneous bonding of AlSi12 powder with a CFRP substrate. Since the thermal properties of the dissimilar materials inhibited a direct build-up of AlSi12 on the CFRP surface, an AlSi12 foil was used as an interlayer for thermal shielding and improved wettability. Within this optimized substrate set-up, the feasibility of hybrid joining via LPBF was demonstrated, while the following challenges were identified for process implementation:

- LPBF led to thermally induced stresses in the AlSi12 volume and the AlSi12 foil, as well as shear stresses at the AlSi12 foil/adhesive interface, which caused adhesive failure and debonding, even at a lower laser energy input.
- In addition to the porosity of the AlSi12 powder layers, VED also correlated with the development of thermally induced stresses and the resulting substrate failure, setting a VED limit ≤ 14 J/mm^3^ for LPBF.
- Since the AlSi12 volume density increased with increasing VED, the opposing trends for a reliable bond strength defined a narrow process window of VED = 11–13 J/mm^3^.

Within this process window, the bonding strength of the hybrid joints was quantified using a novel mechanical testing method, leading to the following conclusions:

- VED ≤ 12 J/mm^3^ generated insufficient welds at the AlSi12 volume/foil interface, where an adhesive fracture occurred at shear strength values τ < 20 MPa.
- VED ≥ 13 J/mm^3^ demonstrated sufficient fusion of the AlSi12 powder with the AlSi12 foil, constantly reaching shear strength values τ > 20 MPa. However, minor AlSi12 foil debonding at the AlSi12 volume edges led to a multi-axial stress state within the foil, resulting in its cohesive failure.

In summary, the results for the bond strength of the presented LPBF-based hybrid joints are not only comparable to those of other innovative joining methods from the literature, but can also compete with those of commercial joining processes. As the feasibility of the presented LPBF-based joining method is demonstrated in this study, future work will address the production of innovative and complex joint geometries with internal features, such as cable or cooling channels. Future upscaling should therefore consider the thermal process limitations and their dependence on the bond dimension and geometry. Moreover, based on the flexibility of the AlSi12 foil integration during CFRP production, bond design is only limited by the accessibility of the joining area for powder application and laser processing.

## Figures and Tables

**Table 1:** Specifications of the LPBF equipment: TruFiber 1000 laser and Scanlab intelliSCAN 30 optical system.

Characteristic	Unit	TruFiber 1000 Laser
Wavelength	λ in nm	1075
Maximum laser power	P_max_ in W	1000
Beam quality	M^2^	<1.3
Pulse duration	T_P_ in µs	20–continuous wave
Focal length	*f* in mm	255
Focal diameter	*d_spot_* in µm	46
Rayleigh length	*z_RL_* in mm	1.2

**Table 2:** Laser powder bed fusion (LPBF) parameters for substrate set-ups A and B.

Parameter	Unit	Substrate Set-Up A	Substrate Set-Up B
Laser beam diameter	B_d_ in µm	200	200
Layer thickness	L_t_ in µm	100	100
Hatch distance	H_s_ in µm	46	200
Scan velocity	V_s_ in mm/s	400	400–700
Laser power	P in W	45–65	160–190
Volumetric energy density	VED in J/mm^3^	7–15	11–24

## Data Availability

The data presented in this study are available on request from the corresponding author.
